# Cold Hardiness Dynamics and Spring Phenology: Climate-Driven Changes and New Molecular Insights Into Grapevine Adaptive Potential

**DOI:** 10.3389/fpls.2021.644528

**Published:** 2021-04-29

**Authors:** Valeria De Rosa, Giannina Vizzotto, Rachele Falchi

**Affiliations:** Department of Agricultural, Food, Environmental, and Animal Sciences, University of Udine, Udine, Italy

**Keywords:** *Vitis vinifera*, chilling requirement, deacclimation, budburst, spring frost, gene expression, demethylation

## Abstract

Climate change has become a topic of increasing significance in viticulture, severely challenged by this issue. Average global temperatures are increasing, but frost events, with a large variability depending on geographical locations, have been predicted to be a potential risk for grapevine cultivation. Grape cold hardiness encompasses both midwinter and spring frost hardiness, whereas the avoidance of spring frost damage due to late budbreak is crucial in cold resilience. Cold hardiness kinetics and budbreak phenology are closely related and affected by bud’s dormancy state. On the other hand, budbreak progress is also affected by temperatures during both winter and spring. Genetic control of bud phenology in grapevine is still largely undiscovered, but several studies have recently aimed at identifying the molecular drivers of cold hardiness loss and the mechanisms that control deacclimation and budbreak. A review of these related traits and their variability in different genotypes is proposed, possibly contributing to develop the sustainability of grapevine production as climate-related challenges rise.

## Introduction

Climate change is a proven reality whose consequences on human activities and natural systems have reached an undeniable magnitude all around the world (IPCC, 2014). Global mean surface temperatures are predicted to increase by 0.3–4.8°C by the end of the 21st century, depending on the trend of anthropogenic greenhouse gas emissions, compared to the reference time-frame 1986–2005 (IPCC, 2014). Many plant species are expected to be unable to shift their geographical range quickly enough to keep up with these changes, and production will be negatively impacted if no adaptation occurs. Rainfall changes are likely to differ depending on the region, whereas radiation and extreme weather events are expected to increase (IPCC, 2019). Agriculture and viticulture, in particular, greatly depend on thermal regimen, soil composition, and water availability, in terms of fruit yield and metabolite composition (van Leeuwen and Darriet, 2016). Grapevine holds great economic value as it can be used fresh (table grape) or dry (raisin) and for winemaking (Delrot et al., 2020). Climate variations in wine-producing regions induce the so-called “vintage effect,” the year-to-year variations in yield, quality, and typicity (van Leeuwen and Darriet, 2016). Grape berry composition also depends on “terroir,” defined as the complete natural environment in which a wine is produced, in which climate plays a major role, with the interplay of human activity (Delrot et al., 2020; Santos et al., 2020). Grapevine phenology and fruit ripening are greatly affected by temperature conditions. Berry composition is key in determining the subsequent quality of wines. The increase in temperature has been shown to cause a rise of berry sugar concentration (Coombe, 1987), whereas some secondary metabolites, such as malic acid or anthocyanins (Kliewer and Torres, 1972), are negatively affected. Higher temperatures produce an advance of phenology, causing earlier harvest dates (van Leeuwen and Darriet, 2016) and decoupling sugar and phenolic compound accumulation at maturity, thus leading to unbalanced wines (Sadras and Moran, 2012; Bonada et al., 2015). High temperatures during the final stages of berry growth, together with high precipitations, can also be the cause of cracks and rots (Molitor et al., 2016). Although rainfall tendencies are difficult to predict, the increase in evapotranspiration caused by temperature increase will cause plants to experience water stress even when rainfall does not directly decrease (van Leeuwen and Darriet, 2016).

The new climate change scenario will lead to increasing difficulty in the production of traditional wines in their areas of origin if no adaptation occurs. Therefore, adaptation measures are necessary as wine quality greatly depends on ripening conditions (Bonada et al., 2015), which in turn are a direct consequence of the timing of several phenological phases starting with budbreak.

Although the impacts of climate change are expected to be diverse in different wine-making regions (Santillán et al., 2019) and among cultivars with different phenological rhythms (McIntyre et al., 1982), several adaptation practices may be able to cope with the short-term effects of climate change and maintain wine typicity, and new training systems could be developed for the middle term (Duchêne, 2016). Remarkably, several variations in training systems and cultural practices have been adopted and tested in recent times with the aim to lower the risk of freezing damage in spring. Trimming, hedging, or pruning has been evaluated in order to mitigate the short-term impacts of climate change (Herrera et al., 2015; Frioni et al., 2016; Palliotti et al., 2017; Abad et al., 2019). In the past, late winter pruning was shown to be effective in delaying bud burst in cool climate areas (Trought et al., 1999), although it could not be applied for grapevine grown in different environments, in which both yield increase (Friend and Trought, 2007) and loss (Frioni et al., 2016) were observed. Recently, a double-pruning approach has shown a potential budburst delay of up to 4 weeks, depending on the timing of the second pruning (Palliotti et al., 2017). As regards the direct avoidance of spring frost damage, several methods, encompassing active and passive types, have been used in the past (Liu and Sherif, 2019). Active approaches include the use of wind machines and helicopters to force the warmer air toward the ground, or heaters and irrigation, to exploit the fusion heat of water. Efficacy of such methods depends greatly on external factors and cannot guarantee a complete avoidance of damage. Moreover, these approaches are costly and environmentally unsustainable and require coordinated action by growers to avoid the rise of production costs and to ensure the effectiveness in the short term (Unterberger et al., 2018). Additionally, the application of chemicals (e.g., Amigo oil, FrostShield, and ProTone) and plant growth regulators (i.e., ethephon) has been shown to delay budbreak, although these results remain inconsistent (Qrunfleh and Read, 2013; Centinari et al., 2018; Kovaleski and Londo, 2019; Liu and Sherif, 2019; Wang and Dami, 2020).

In this context, the genetic improvement of grapevine has been taken into consideration to cope with the effects of climate change in the long run. Cultivated grapevines all around the world are usually grafted, and this adds a layer of complication to the understanding of plant–environment interactions. Moreover, the communication between scion and rootstock is often unclear or unexplored as the connection that is immediately established at grafting may evolve as the plant ages (Delrot et al., 2020). Therefore, despite the numerous aspects to consider, the investigation of unexploited varieties in germplasm collections, for both rootstock and scion, could be an interesting opportunity, strengthened by the continuous evolution of sequencing technologies and gene-mapping approaches. Efficient phenotyping methods also need to be developed to assess the effectiveness of varietal selection and the plasticity of the phenotype in different scion–rootstocks combinations (Warschefsky et al., 2016). As an example, recent studies have shown that different clone–rootstock combinations can influence and level cold hardiness differences among cultivars (Hébert-Haché et al., 2020). However, the possibility that the variability within clones of the *Vitis vinifera* species might be insufficient to compensate the phenological shifts caused by climate change must be contemplated; the need to introduce new varieties with the abandonment of the traditional ones will eventually arise if no measure is taken (Duchêne, 2016). Moreover, in addition to the already existing varieties, new ones could be generated through traditional breeding approaches or even genetic engineering. In any case, the comparison and analysis of different *Vitis* species could, first, help in clarifying the molecular regulators and drivers of cold hardiness, deacclimation, and budbreak and, second, allow the identification of targets to optimize clone selection and breeding efforts.

In this review, spring frost frequency and trends for different geographical regions are reported, together with the recent findings about the potential pathways involved in cold deacclimation and budbreak. We aim to provide an update on current status of research regarding the effects of climate change on grapevine phenology, with a focus on cold hardiness dynamics, budbreak, and the key molecular players involved in these processes. This will hopefully help in developing new ways to face current and future climate-related contingencies to allow berry ripening and harvest to be achieved in favorable conditions.

## Effects of Climate Change on Grapevine Phenology

Several studies have assessed the impact of climate change on grapevine phenology and viticulture in the past and in the present (Biasi et al., 2019), and numerous models have been tested to predict future consequences (Caffarra and Eccel, 2011; Bonfante et al., 2017; Alikadic et al., 2019; Costa et al., 2019; Ramos and de Toda, 2020). Agroclimatic indices are considered more reliable than individual climatic variables to describe climate change effects (Santos et al., 2020); these tools allow to closely follow and simulate plant development in different scenarios and can be used to evaluate the potential of different areas for viticulture (Molitor et al., 2014; Blanco-Ward et al., 2019). Redistribution of wine production within continents is a likely perspective, and the change in viticultural suitability for different geographic regions has been calculated, showing agreement among 17 global climate models. Wine-producing regions will possibly decrease by 2050 (mainly in Mediterranean climate area), whereas expanding suitability has been predicted an increase for New Zealand, western North America, and Northern Europe (Hannah et al., 2013).

However, commonly bioclimatic indices used in viticulture (e.g., Huglin Index, Winkler Index, Dryness Index, Cool Night Index) are arguably replaced by dynamic crop models (e.g., STICS, BRIN), which combine several indices and integrate phenotype, soil, weather data, and management practices into a more comprehensive picture (Cortázar-atauri et al., 2009; Moriondo et al., 2013; Fraga et al., 2016). Heat requirements, determined in terms of growing-degree days (GDD), represent the climatic constraint that allows grape to successfully complete its annual cycle when met. Distinct phenological phases need different climatic conditions to take place (e.g., release from ecodormancy; Ruml et al., 2016). Higher temperatures lead to an acceleration of plant development, being a potential cause of premature loss of bud cold hardiness (Pagter and Arora, 2013; Londo and Kowaleski, 2017; Kovaleski et al., 2018). In fact, early events such as budbreak and flowering have been shown to be the most sensitive to temperature-driven variations as compared to later phases (Jones et al., 2005). This increases the chances of vulnerable green tissues to be exposed to late spring frost events, which have been known to be the cause of great yield losses in the past (Gu et al., 2008). The timing of budbreak is strictly linked to the end of dormancy, a genetically programmed state of self-arrest in which the bud stops its development to avoid breaking at unfavorable times (Lang et al., 1987; Horvath et al., 2003). Whether the risk of damage due to spring frosts is globally increasing is up to debate, although recent reports suggest the relevance of this phenomenon in several locations (Augspurger, 2013; Ma et al., 2018; Sgubin et al., 2018). Effects are expected to vary, depending on the geographical position, and changes in water availability need to be taken into account together with temperature variations. Great attention has been always given to budbreak timing as early dormancy release in cold winter regions can cause significant crop losses, and frost-protecting measures represent a notable cost for producers. To the contrary, warmer regions can be affected by low rates of budburst and lower productivity due to insufficient chilling during winter, making the use of artificial dormancy-breakers a necessity. Vineyards located in southern Europe (e.g., Italy, Spain, Portugal) are expected to experience increased water stress conditions especially during summer, leading, together with warming, to yield and quality reduction (Fraga et al., 2017; Santillán et al., 2019). Severe dryness is, in fact, the main reason impairing viticulture suitability in these areas. On the other hand, increasing average temperature has been predicted to have positive outcomes on winemaking regions in central and Western Europe and to allow the extension of viticultural areas in the north and east (Gaal et al., 2012; Cardell et al., 2019). This will favor the introduction of new currently inaccessible varieties in colder areas, as frost is expected to decrease and optimal ripening temperatures to be reached (e.g., Northern Europe, North America; Santillán et al., 2019); moreover, wine-producing suitable areas are expected to develop up to the 55°N by 2070 (Fraga et al., 2016).

### Cold Hardiness Variations

Dormancy encompasses endodormancy, determined by internal factors, which allows buds to cold acclimate and reach a state of hardiness to survive freezing temperatures during winter. Cold acclimation is a process in which physiological, biochemical, and epigenetic changes driven by cold temperatures confer freezing tolerance (Wisniewski et al., 2018). Exposure to chilling temperatures, with difference depending on cultivar (Anzanello et al., 2018), is required to resume bud responsiveness to environmental signals and avoid growth start if mild temperatures occur during winter (Rohde and Bhalerao, 2007). Internal signals also prevent growth resumption in late summer or early autumn, which would cause the death of the bud in unfavorable environmental conditions (Lang et al., 1987; Horvath et al., 2003).

The productivity of grapevine and temperate plants is related to the capability of buds, both reproductive and vegetative, to tolerate freezing temperatures. Cold hardiness correlation with winter temperatures has been measured (Kovaleski et al., 2018). In general, sudden or recurring warm spells in winter can endanger the survival of woody perennials to freezing temperatures because the deacclimation process, during which cold tolerance is lost, is relatively fast (Pagter and Arora, 2013). Although deacclimation and acclimation cycles seem possible and efficient in several herbaceous plants (Vyse et al., 2019), it appears diverse for woody perennials with cold acclimation being restored only in part (Shin et al., 2015). Various grapevine species have been shown to be differently responsive to temperature variations during dormancy, likely related to the dissimilar chilling requirements that allow the transition from endodormancy to ecodormancy, at distinct timings. In addition, maximal cold hardiness is not reached automatically, and a cold sustained winter is needed (Londo and Kowaleski, 2017). Depending on the species, grapevine buds’ cold hardiness can reach temperatures below −30°C (Londo and Kowaleski, 2017). However, once buds begin to swell and deharden during the deacclimation process, their freezing tolerance quickly reduces, and the observed advancements in phenological timings may possibly increase the exposure of vulnerable plant structures to late frost events.

### Spring Frost Risk

Late spring frosts have often resulted in great damage to cultivated fruit trees and in important economic losses (Gu et al., 2008; Marino et al., 2011; Ault et al., 2013; Vitasse and Rebetez, 2018). In the bigger picture, these phenomena can alter the ecosystem and evolution of entire populations because of competition among species and parasite opportunism (Inouye, 2000; Reineke and Thiéry, 2016). As previously stated, the vulnerability of plant structures to freezing temperatures differs, depending on their level of cold hardiness, which varies seasonally, and on their intrinsic ability to sustain lower temperatures. Green tissues, flowers, and fruit are, in fact, significantly more susceptible to lower temperatures than wooden tissues as their hydration levels are considerably higher, and their supercooling capabilities lower (Fennell, 2004). Budburst and leafout have been delineated as the most critical, as several trees have been shown to be the most vulnerable at that specific time (Vitasse et al., 2014; Lenz et al., 2016). Moreover, a lower temperature stability is expected during winter in the future, which will require the use of cultivars with a lower response to so-called “false springs” (Londo and Kowaleski, 2019). A “false spring” can be empirically defined as a period of warm temperatures with premature rapid vegetative growth, followed by a freeze (Gu et al., 2008; Ault et al., 2013); several mathematical approaches to evaluate these phenomena have been attempted (Marino et al., 2011). Freezing temperatures following a “false spring” can culminate in more serious damage, which affects photosynthetic tissue and reproductive tissue alike with consequences spread on multiple years of development (Carmona et al., 2008). In general, the influence of climate change on late frost events frequency and distribution remains unclear, and whether risk is increasing for temperate trees remains up for debate. The analysis of remote-sensing data showed that frost day in which the temperature drops below 0°C during the growing season have increased in the Northern Hemisphere (Liu et al., 2018). Concerning Europe, phenological and climate records were used to analyze the evolution of spring frost risk as regards several tree species, between 1950 and 2013, with a focus on determining variations in the frequency of the phenomenon (Ma et al., 2018). These results showed that species whose phenology is more responsive to temperature increases tend to experience a higher risk of being subjected to frost occurrences and damage. Maritime areas in Europe were also more exposed to frost compared to continental ones (Ma et al., 2018). Besides, high-altitude areas could experience decreased risk as the rate of warming seems to be amplified with elevation (Pepin et al., 2015). The effects of late frosts on the distribution of grapevine in Europe were analyzed (Leolini et al., 2018). The results, simulated under future scenarios, described in the AR5 IPCC (2014) report, show that budbreak and flowering advancement are more pronounced in Northeastern Europe compared to the Southwest. The simulations showed that changes in the phenology stages of grapevine might expose it to higher frequency of extreme events, with the effects being strictly linked to the phenological cycle of the considered variety (Leolini et al., 2018). An increased risk of spring frost damage is also predicted in several regions of France, supported by two budburst day simulation models (Sgubin et al., 2018). Similarly, a high probability of spring frost damage for several woody species in Illinois (United States) was reported, by integrating field observations of temperature, phenology, and frost damage over long timeframes (Augspurger, 2013). “False spring” occurrences were reviewed across the United States over the 1920–2013 interval by taking into consideration the trends of vegetation start dates, spring freezes, and a sensitivity analysis, which indicated a decrease in spring frost exposure (Peterson and Abatzoglou, 2014), pointing out distinct tendencies for different geographical locations.

## Long-Term Resilience to Climate Change

### Breeding Approaches

Passive spring frost damage avoidance approaches are used preemptively and are suited to work on the long-term and include breeding and selection of new fitter varieties (Liu and Sherif, 2019). Traditional breeding approaches have been successfully used in the past to select new cultivars with characteristics of economic interest and in a perennial crop such as grapevine the entire traditional breeding procedure and evaluation process can take many years to be completed (Eibach and Töpfer, 2015). As cultivated grapevines are propagated clonally to fix and maintain specific production parameters, somatic variations that can accumulate during clonal propagation are almost the only source of genetic diversity (Carbonell-Bejerano et al., 2017; van Houten et al., 2020), greatly lower than intervarietal diversity (Roach et al., 2018). Clone collections exist and are available worldwide and represent a source that should be accessed to search for interesting genotypes (Duchêne, 2016). A possible adaptation for the current grape-growing areas should consist in the selection of varieties with a later ripening period; such varieties can be obtained from germplasm collections or through breeding processes (Duchêne et al., 2012).

Fruit trees must fulfill a chilling requirement to transition from endodormancy to ecodormancy, a phase of dormancy in which buds are responsive to growth-promoting conditions. The amount of chilling hours required to do so depends on the genotype, and genotypes that require less chilling have been shown to deacclimate earlier. In any case, the models describing winter chill accumulation are purely empirical or based on experiments in controlled conditions, and the physiological processes occurring in plants during winter are still poorly understood (Luedeling and Brown, 2011). The most popular chilling-hours accumulation models estimate effective chilling temperatures to be included in the 0–7.2°C interval (Dokoozlian, 1999), although different models attribute varying effectiveness to specific temperatures or even negative impacts of higher temperatures on previously accumulated chill (Darbyshire et al., 2011). The widely applied and possibly most accurate Dynamic Model also suggests that the same temperatures might have inconsistent effectiveness, depending on which time of the season they are registered, making it difficult to transfer available information from one location to another (Luedeling, 2012). Cultivated grapevines are generally considered low-chilling-requiring species compared to other woody perennials; however, chilling requirements can differ significantly in high‐ and low-chill varieties and fast‐ or slow-burst phenotypes (Londo and Johnson, 2014). Production located at higher latitudes could benefit from the use of grapevines characterized by higher chilling requirements and slower budburst rates, which would allow lowering the risk of spring frost damage (Londo and Johnson, 2014). Wild grapevines presented a continuous range of chilling requirements and budburst rates, making them an interesting source of variability. In detail, *Vitis amurensis*, *Vitis labrusca*, and *Vitis riparia* were classified as low-chill and fast-burst species, whereas *Vitis rupestris*, *Vitis aestivalis*, and *Vitis vulpina* showed higher chilling requirements (>1,000 h) and longer budburst timings (>14 days). Different latitudes were also proposed as seemingly having an adaptive effect. In fact, North-distributed genotypes (*V. riparia, V. labrusca*, and *V. amurensis*) were all classified as low-chill, fast-bursting species. On the contrary, southern varieties (*V. aestivalis*, *V. cinerea*, *V. rupestris*, and *V. vulpina*) were all characterized by higher chilling requirements and slower budburst timings (Londo and Johnson, 2014).

Hybrid crosses were shown to allow lowering the deepest level of cold hardiness, although this could also introduce enhanced midwinter responsiveness in areas where climate warming produces mild winter temperatures (Londo and Kowaleski, 2019). Deacclimation rates were also observed to be much faster in wild varieties *V. riparia* and *V. amurensis*, commonly used by breeders to increase freezing tolerance in cultivated varieties. This could contribute to increased risks of deacclimation during warmer winters and of spring frost damage (Kovaleski et al., 2018). These phenomena could be explained by the evolutionary necessity of these varieties to develop rapidly during short growing seasons typical of their area of origin (Ferguson et al., 2014). Paradoxically, this would make the varieties with the deepest levels of cold hardiness also the most vulnerable to spring frost damage (Ferguson et al., 2014), and considering the observed advancement of spring phenology, winter-hardy varieties could display unwanted phenotypes. For these reasons, focusing breeding efforts on the production of delayed growth-start cultivars could be an alternative favorable approach. A prerequisite for this strategy is the gaining of a comprehensive understanding of the biochemical and molecular mechanisms responsible for dormancy establishment and release in grapevine buds.

Rootstocks are traditionally used to protect scions from soil-borne pests and to improve tolerance to various abiotic stresses; however, their effects on the entirety of the plant often remain obscure (Ollat et al., 2016). The breeding of new rootstocks needs to be considered as a long-term strategy to cope with the consequences of climate change as the substitution of traditional scions with new ones is not going to be accepted as easily. The genetic background of commonly used rootstocks can be difficult to understand as their heritage is often mixed (Poczai et al., 2013), but efforts to improve breeding by enhancing the knowledge of genetic markers have been attempted in recent years (Migliaro et al., 2019; Riaz et al., 2019). This information is important and needs to be exploited to improve marker-assisted selection (MAS) of new rootstocks, as their influence on scion signaling molecules, response to several stresses, and even berry quality has been observed (Tramontini et al., 2013; Pagliarani et al., 2017; Martin et al., 2020; Zombardo et al., 2020). Moreover, rootstocks can alter scion development rate possibly because of their different abilities to take up nutrients and water from the soil (Zhang et al., 2016). Additionally, messenger RNA molecules and hormones have been reported to pass through the graft site in a possibly environment‐ and genotype-dependent manner (Nikolaou et al., 2000; Yang et al., 2015). Putative rootstock effects on grapevine phenology and, in particular, on its heat requirements have also been described (Miele, 2019).

A great boost in breeding effort can be attributed to the identification of molecular markers, the introduction of genetic mapping, and genotype–phenotype associations, considerably facilitated by the release of the complete sequence of the *V. vinifera* genome (Jaillon et al., 2007; Velasco et al., 2007). MAS can help the identification of sequences with different genetic backgrounds, aiding the potential exploitation of wild *Vitis* species carrying traits of interest (Daldoul et al., 2020).

### Molecular Mechanisms Involved in Deacclimation and Budbreak

Monitoring dormancy status of the bud in real time appears really challenging, because of the absence of visual changes during the bud dormancy cycle (Or, 2009), and the use of GDD as a proxy for spring phenology is not always reliable. Therefore, a better knowledge base of the physiological mechanisms underpinning dormancy induction and release can be an important part of predicting the potential effects of global warming on grapevine. A strict correlation between budbreak and loss of winter cold hardiness (deacclimation) has been recently hypothesized, pointing out that a temperature-controlled interplay underpins these phenological changes (Kovaleski and Londo, 2019).

In this context, recent advances in the understanding of cold hardiness and spring budburst mechanisms may contribute to enhance the sustainability of viticulture, especially when acute cold weather events are expected to increase (Kovaleski and Londo, 2019). On the other hand, traditional breeding is also empirical and requires a deep knowledge of the physiological characteristics of the selected cultivars in past and present cultivated areas. Recently introduced molecular approaches allowed new methods of “molecular breeding” to be applied, allowing speedier and refined crosses (Delrot et al., 2020).

Unfortunately, phenological traits, such as budburst, are often regulated by many quantitative trait loci (QTLs), which are highly responsive to environmental factors. For this reason, the mapping and cloning of genes related to phenological traits are really challenging, and the reproducibility of these QTLs remains low (Delrot et al., 2020).

Recently, several works have identified QTLs associated with budbreak. For example, two independent QTLs on chromosomes 4 and 19 were identified using a genetic map build with microsatellites markers on varieties Riesling and Gewurztraminer (Duchêne et al., 2012). The WRKY transcription factor *Vv*WRKY3 was found within the confidence interval on chromosome 19; a similar transcription factor, *At*WRKY2 from *Arabidopsis*, was shown to mediate ABA (abscisic acid) control on seed germination (Jiang and Yu, 2009). Moreover, several genes encoding glutathione S-transferases (GSTs) were also identified on both chromosomes 4 and 19. Increased levels of expression of these genes were registered after both HC (hydrogen cyanamide) application (Or, 2009), a dormancy-breaking agent, and after the natural fulfillment of chilling requirements (Pacey-Miller et al., 2003). Similarly, simple sequence repeats (SSRs) and single nucleotide polymorphisms (SNPs) were used to map another QTL related to budburst on chromosome 15, overlapping on QTLs related to veraison (Grzeskowiak et al., 2013). Genes on chromosome 15 included several transcription factors involved in bud and fruit development (Grzeskowiak et al., 2013).

With regard to cold hardiness control, the progeny resulting from the cross between cold-vulnerable cv. Cabernet Sauvignon and the cold-tolerant hybrid Zuoyouhong was used for the construction of a high-density genetic linkage map on which cold hardiness-related QTLs were mapped (Su et al., 2020). Six QTLs located on chromosomes 2, 3, and 15 were identified, and four cold-responsive candidate genes were proposed. In detail, a dehydration-responsive protein containing a *cis*-DRE (dehydration responsive) element was identified. CRT (C-repeat)/DRE elements, containing a core CCGAC sequence designated as C-repeat, are present in single or multiple copies in the promoter regions of plant COR (cold-responsive) genes, which are induced by low-temperature exposure (Stockinger et al., 1997). The COP9 signalosome (CSN) subunit 1 was also individuated; CSN was shown to be required for the expression of *COR* genes in *Arabidopsis* (Schwechheimer et al., 2002). Additionally, an RRM (RNA recognition motif)–containing protein was found to be putatively involved in cold hardiness as well. RRM modules were found in cold-responsive RNA-binding proteins from cyanobacteria (Maruyama et al., 1999). Lastly, a MYB-related gene’s expression was also reported to be enhanced by cold exposure. Its overexpression in *Arabidopsis* was previously shown to confer increased tolerance to cold (Sun et al., 2018).

Transcriptomic tools have led to new insights into the gene expression processes that take place in dormant tissues. Dormancy release is regulated by a multitude of independent genes whose mechanisms of action are still unclear, together with their conservation among species (Table 1). Growth resumption happens simultaneously with cold deacclimation, although most hardiness is already lost when new tissue is visible (Kovaleski and Londo, 2019). Growth start is also subordinate to the fulfillment of the chilling requirement and the transition from endodormancy to ecodormany, in which the bud becomes sensitive to favorable environmental conditions. CBFs/DREBs (C-repeat binding factors/dehydration responsive element binding) are important cold-response regulators stimulated by low temperatures. These transcription factors act as a part of a signaling cascade in which they are induced by ICEs (inducers of CBF expression) and activate COR genes by binding to the CRT/DRE *cis*-elements in their promoter regions and thus conferring freezing tolerance to the plant (Chinnusamy et al., 2010; Thomashow, 2010). Another cold-responsive transcription factor, bHLH, was characterized in both *V. vinifera* cv. Cabernet Sauvignon and wild *V. amurensis* with a proposed putative regulatory role in cold stress response in a CBF-dependent way (Xu et al., 2014). Changes in expression levels and timing of *VvbHLH* and *VabHLH* were observed, possibly caused by differences in the *cis*-regulatory elements in their sequence (Xu et al., 2014). CBFs/DREBs have been identified in several woody species as well as *Arabidopsis*, and their functions are highly conserved (Wisniewski et al., 2014). Several CBFs/DREBs are known in grapevine (Xiao et al., 2006; Tillett et al., 2012; Rubio et al., 2019a) and show increased mRNA expression following exposure to freezing temperatures (Xiao et al., 2006, 2008). The most well-known targets of CBFs/DREBs are DHNs (dehydrins), part of the LEA (late embryogenesis abundant) proteins. DHNs accumulate during dormancy induction and cold acclimation and protect cells from dehydration damage (Wisniewski et al., 2014). Four grape DHNs have been identified (Yang et al., 2012). DHNs were reported to be differently expressed in wild *V. riparia* and in cultivated variety Chardonnay following cold exposure (Xiao and Nassuth, 2006). Increased freezing tolerance is also observed in case of *VvCBFs* overexpression (Tillett et al., 2012). Moreover, the synergistic effect of low temperatures and ABA application in stimulating the expression of CBFs/DREBs in grapevine dormant buds has been recently assessed (Rubio et al., 2019a). ABA has a key role in plant dormancy regulation as ABA variations have been correlated to different degrees of seed dormancy (Nambara et al., 2010). ABA’s role in bud dormancy in woody perennials has been hypothesized, although the regulation mechanism is complex and is still obscure. Recently, several studies showed that the highest levels of ABA were reached at the maximum depth of dormancy and started decreasing at the end of endodormancy in grapevine buds (Kovaleski and Londo, 2019; Rubio et al., 2019b). ABA was also observed to promote starch synthesis in dormant buds, thus promoting their sink capacity and regulating dormancy depth this way (Rubio et al., 2019b). Changing ABA balance in the buds is also the mechanism by which dormancy-breaking agents, such as HC, seem to accomplish their effect (Zheng et al., 2015; Rubio et al., 2019b). In detail, the budbreaking effect of HC in grapevine was reported to be exerted by the stimulation of the ABA-degrading enzyme ABA 8′-hydrolase (A8H), encoded by the *VvA8H-CYP707A4* gene (Zheng et al., 2015). A8H and ABA catabolite increase was also observed during natural dormancy release (Zheng et al., 2015). Moreover, the reversible ability of ABA to prevent loss of cold hardiness and deacclimation after several days of prolonged application on grapevine buds was observed (Kovaleski and Londo, 2019). Together, these results suggest an important role of ABA in endodormancy maintenance and dormancy release, but not in its induction. More recent studies showed that transgenic vines overexpressing *VvA8H-CYP707A4* show both a higher catabolism of ABA and an enhancement of budbreak. Hypoxia and ethylene, which are both considered dormancy release stimulants, enhance the expression of *VvA8H-CYP707A4* (Zheng et al., 2018a). Multiple studies have shed light on the role of other hormones in dormancy release and budbreak; for example, a recent work focused on the expression of several genes involved in the gibberellin (GA) biosynthetic pathway and the interaction of GAs with cytokinins (CKs) in grapevine buds (Zheng et al., 2018b). Although further studies are required, the authors propose an inhibitory effect of GA on budbreak that would give account of the low levels of this hormone registered during dormancy. Authors also hypothesize that this inhibition results from the antagonistic effect of GAs on CK responses, which are required for bud meristem reactivation; only following meristem activation higher levels of GA could be required to sustain growth and budbreak (Zheng et al., 2018b). In addition to this, the effects of cold temperatures on the concentration of salicylic acid (SA) and the expression of genes in its biosynthetic pathway in dormant grapevine buds were also explored (Orrantia-Araujo et al., 2020). Buds exposed to longer periods of chilling hours showed a higher content of endogenous SA once transferred in forcing conditions. The expression of genes *ICS2* (isochorismate synthase 2), *NPR1* (nonexpressor of PR genes 1) and *WRKY70* showed variations in buds subjected to cold treatment compared to control ones. ICS2 takes part in the biosynthesis pathway of SA, NPR1 is a master regulator of SA-mediated defense signaling, and WRKY70 participates in both positive and negative regulation of SA signaling. These results indicate that cold accumulation could stimulate the synthesis of SA in grapevine buds and introduce the possibility of a role of SA-mediated defense signaling in bud dormancy release (Orrantia-Araujo et al., 2020).

**Table 1:** Genes with putative involvement in cold deacclimation and budbreak regulation.

Gene	Physiological role	Reference
*CBFs/DREBs*	Low-temperature response	Xiao et al., 2006
*bHLH*		Tillett et al., 2012
		Xu et al., 2014
		Rubio et al., 2019a
		Su et al., 2020
*VpERF2*		Zhu et al., 2013
*VpERF3*		Gibbs et al., 2014
*VvA8H*	ABA regulation	Duchêne et al., 2012
*VvWRKY3*		Zheng et al., 2015
		Zheng et al., 2018a
*VvICS2*	Defense mechanisms	Zheng et al., 2018b
*VvNPR1*		Orrantia-Araujo et al., 2020
*VvWRKY70*		
*VaCPK20*	Ca^2+^ transport	Dubrovina et al., 2013
*CNGCs*		Kovaleski and Londo, 2019
*FAD5*	Membrane fluidity	Kovaleski and Londo, 2019
*GSTs*	Hypoxia response and oxidative stress	Duchêne et al., 2012 Grzeskowiak et al., 2013
*ERF-VIIsRBOHF*		Meitha et al., 2018
		Kovaleski and Londo, 2019
*EBB1*	Growth resumption	Busov et al., 2016
*DMLs*	Chilling-responsive demethylation	Conde et al., 2017
		Shangguan et al., 2020

The discovery and characterization of the *EBB1* gene with a role in shoot growth resumption after winter have been carried out both in *Populus* (Yordanov et al., 2014) and in peach, where RNA-seq analysis confirmed that EBB1 is involved in budbreak by taking part into the regulation of several pathways that act synergistically and involve hormones, cell division, and cell wall modifications (Zhao et al., 2020). The conservation of this AP2/ERF family transcription factor was evidenced by the identification of several homologs in various woody perennial species, among which also is *V. vinifera* (Busov et al., 2016). Consistently with the EBB1 expression in *Poplar*, *VvEBB1* resulted greatly downregulated during dormancy and upregulated before budbreak.

It is well-known that genomic DNA methylation is a mechanism that influences gene expression. In plants, a subgroup of DNA glycosylase-lyases, known as DEMETER-LIKE DNA demethylases (DMLs), can actively demethylate DNA and have been shown to be involved in abiotic stress responses in *Arabidopsis* (Le et al., 2014), developmental transitions in tomato (Liu et al., 2015), and nodule development in *Medicago truncatula* (Satgé et al., 2016). A *Populus trichocarpa* DML, *Pta*DML10, was proposed to be responsible for DML-mediated demethylation at the shoot apical meristem in budbreak regulation (Conde et al., 2017). A loss-of-function analysis confirmed the chilling-responsive demethylation performed by DML10 in proximity to dormancy release. RNA-seq combined with methylome data analysis revealed that the *DML10* gene targets are genetically associated with budbreak (Conde et al., 2017). Moreover, no overlap was found between the targets of DML10-mediated demethylation and EBB1 targets in poplar. This seemingly confirms that these genes act on separate pathways (Conde et al., 2017). No evidence on the role of DML genes on grapevine dormancy release currently exists, although several DML demethylases have been identified (Shangguan et al., 2020).

Additionally, regulated hypoxia has been found to be a development signal in several stages of plant life (Gibbs et al., 2014; Abbas et al., 2015), and many responses to hypoxia are regulated by group VII of ethylene responsive transcription factors (ERF-VIIs) (Gibbs et al., 2014). For these reasons, the role of oxygen-dependent signaling in transcriptional and metabolic reactivation during budburst in grapevine was investigated (Meitha et al., 2018). The data support that oxygen-dependent signaling through grape ERFs is involved in the transition from dormancy to budburst. Moreover, approximately 20% of grapevine genes presenting a HRPE (hypoxia-responsive promoter element) motif in their promoter were differently expressed in the first 24 h of budburst (Meitha et al., 2018). These results strongly suggest an important developmental function of oxygen-dependent signaling through *Vv*ERF-VIIs in determining timing and coordination of budburst in grapevines. Further support of the role of oxidative stress response pathways in grapevine budbreak regulation is provided by Kovaleski and Londo (2019), proposing the expression of RBOHF (respiratory burst oxidase homolog protein F) as a marker for budbreak. RBOHF is involved in ABA and ethylene signaling through H_2_O_2_ production (Kwak et al., 2003). In addition to this, two ERF genes from Chinese wild *Vitis pseudoreticulata*, *VpERF2* and *VpERF3*, were reported to be involved in abiotic stress response pathways including cold exposure (Zhu et al., 2013). Overexpression studies also pointed out a role of these transcription factors in pathogenesis-related proteins accumulation. Moreover, ABA-dependent expression of *VpERF2* and SA-dependent expression of *VpERF3* were shown through exogenous hormone application on leaves (Zhu et al., 2013).

Recently, dormant buds of several *Vitis* genotypes, belonging to different species, were observed to sense the stimulus for dormancy release and deacclimation simultaneously when put into the same forcing conditions (Kovaleski and Londo, 2019). The observed differences in budbreak timings would then be attributed to the ability of the specific genotypes to restart growth. In fact, temperature sensing is believed to be the first step toward bud growth. Among the first sensors, membrane CNGCs (cyclic nongated ion channels) are very responsive to temperature changes. These nonselective Ca^2+^ channels are placed as very first components of the thermosensing pathways in *Arabidopsis* and *Physcomitrella* (Finka et al., 2012) and possibly have the ability to sense membrane fluidity changes caused by temperature shifts (Finka and Goloubinoff, 2014). Synchronous downregulation of nuclear-localized CNGC15 and FAD5 (fatty acid desaturase 5) was reported, suggesting a role of nuclear Ca^2+^ signaling during dormancy in grapevine buds (Kovaleski and Londo, 2019). A role in cold and water stress response of Ca^2+^ flux sensor *Va*CPK20 (calcium-dependent protein kinase) from wild *V. amurensis* vines was also suggested (Dubrovina et al., 2013).

## Concluding Remarks

**Figure 1:**
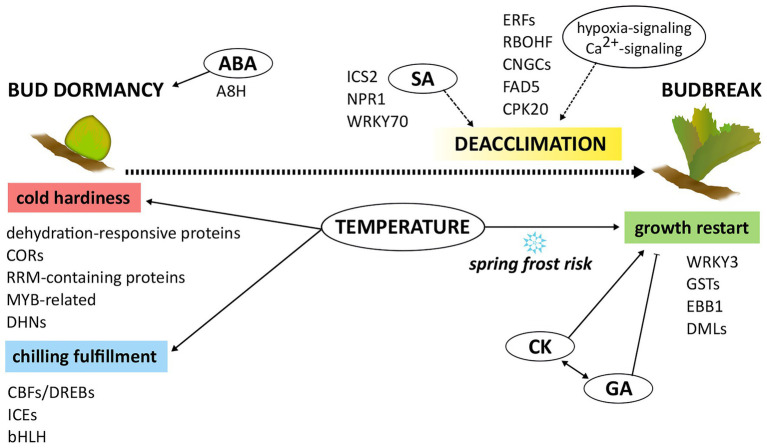
Schematic representation of the current knowledge on the molecular control of bud dormancy-budbreak transition. Temperature plays a key role in influencing both phenological stages. Most of the gene functions involved at each phenological stage are reported, as well as their interplay with other metabolic and hormonal signaling pathways.

Spring frost damage risk cannot be overlooked in the future in several areas of the world, making the identification of effective adaptive measures an issue of the present. Understanding the molecular mechanisms underlying cold hardiness loss/deacclimation and budbreak is essential for improving crop sustainability and adaptation in the future changing climate. The observations gathered so far on cold deacclimation and dormancy release regulation in grapevine outline a very complex scenario in which many pathways are involved (Figure 1). As chilling requirement, deacclimation dynamics, and budbreak timing appear tightly connected, a major regulatory role can be ascribed to temperature-sensing related genes, common among different genotypes. Hormonal interplay, at times synergistic as well as antagonistic or seemingly independent, should also draw great attention as not only ABA’s expected involvement seems ascertained, but also growth reactivation-related, defense-related, and oxidative stress–related hormones putatively perform actively in the regulation of these phenomena. A third valuable and worthy of notice opportunity concerns epigenetics and epigenetic regulators, which add an extra layer of complexity. Defining the extent of the role and significance of each component of this intricate net of regulators requires further studies.

**Figure 2:**
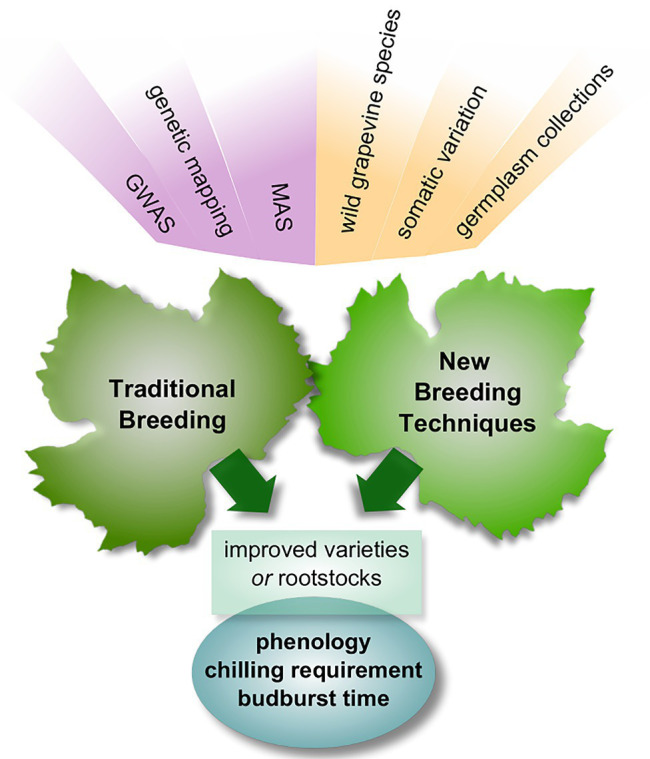
Schematic overview of traditional and new breeding approaches to cope with climate change issues. Natural variability and genetic knowledge are important building blocks of breeding; phenology-related traits are the main target. GWAS, genome-wide association studies; MAS, marker-assisted selection.

Breeding efforts need to focus on the potential of wild *Vitis* varieties to bear favorable traits, starting from changing chilling requirements and budburst rates. In this regard, the accuracy of all most popularly used chilling-hours accumulation models needs to be standardized in order to select varieties suitable to changing conditions is specific areas. An intense application of genetic mapping approaches is required to locate and isolate the genetic *loci* that are responsible for the phenotypic expression of these characteristics so that traditional or new plant breeding techniques can be carried out more swiftly and purposefully (Figure 2). Despite the complexity of the full picture and the uncertainties about the connections among the players, the variety of elements involved allows tackling the problem through a multitude of approaches and should be considered encouraging.

## Author Contributions

All authors listed have made a substantial, direct and intellectual contribution to the work, and approved it for publication.

### Conflict of Interest

The authors declare that the research was conducted in the absence of any commercial or financial relationships that could be construed as a potential conflict of interest.
